# Sediment-redox dynamics in an oligotrophic deep-water lake in Tierra del Fuego: insights from Fe isotopes

**DOI:** 10.1007/s10933-024-00316-0

**Published:** 2024-04-29

**Authors:** Luis Gabriel Ordoñez Rendón, Ina Neugebauer, Camille Thomas, Massimo Chiaradia, Nicolas Waldmann, Daniel Ariztegui

**Affiliations:** 1 Department of Earth Sciences, University of Geneva, rue des Maraîchers 13, 1205 Geneva, Switzerland; 2 Moses Strauss Department of Marine Geosciences, Charney School of Marine Sciences, University of Haifa, Mount Carmel, 3498838 Haifa, Israel; 3 Present Address: Helmholtz-Zentrum Potsdam, Deutsches GeoForschungsZentrum GFZ, Telegrafenberg, 14473 Potsdam, Germany; 4 Present Address: Oeschger Centre for Climate Research, Institute of Geological Sciences, University of Bern, Baltzerstrasse 1+3, 3012 Bern, Switzerland

**Keywords:** Fe speciation, Fe oxyhydroxides, Laminated sediments, Redox fronts, Limnological processes

## Abstract

For long time in the history of Earth, ferruginous conditions governed the oceans. With the rise of oxygen during the Proterozoic era and the subsequent evolution of living organisms, worldwide deposition of iron formations occurred. These sedimentary units reveal the transition into oxic oceans, passing by local and transitory euxinic conditions, especially in coastal shelves. Constraining the iron cycle and the biogeochemical processes occurring in present and past ferruginous basins helps answering some of the question regarding global oxygenation, the evolution of life and past climate changes. Therefore, Fe speciation and Fe isotopes in both Proterozoic and recent sedimentary records have been widely used to reconstruct past basin dynamics and redox conditions in the sediment–water interface. However, sedimentation and early diagenesis can alter paleoredox proxies and their primary climate signals. In this work, we disentangled alteration processes occurring at the redox front below the sediment–water interface of a ventilated deep-water lake (Lago Fagnano, Argentina/Chile). A sequential extraction protocol was applied to characterize two reactive Fe pools: Fe oxyhydroxides and reduced iron. Subsequently, Fe isotopes were constrained to determine the main processes mobilizing Fe. At the redox front, ferric minerals reach a *δ*^56^Fe value of − 1.3‰ resulting from oxidation of dissolved Fe likely following a Rayleigh distillation effect. Dissolved Fe is produced right below via Fe reduction, as shown by the low ferric Fe content. Our observations delineate a redox cycle and a redox horizon undergoing constant upward migration, initiated by regular sedimentation. However, during events of increased rapid sedimentation (e.g., seismites) this dynamic cycle is interrupted inducing full or partial preservation of the Fe-rich redox front. In such case, oxidation of dissolved Fe is interrupted and can be recycled in ferrous minerals, such as Fe monosulfides and amorphous phases with *δ*^56^Fe values down to − 1.7 ‰. These findings have significant implications for the recording of biogeochemical cycles in the geological past, the use of Fe isotopes in freshwater-lake sediments for paleoclimate studies, and the progress of our knowledge regarding the geochemistry of past oceans.

**Supplementary Information:**

The online version contains supplementary material available at 10.1007/s10933-024-00316-0.

## Introduction

Lake sediments are perfect archives for studying climate evolution at different time scales. The reason is that lacustrine systems are particularly sensitive to climate–induced factors, such as moisture and wind. These can considerably affect basin dynamics and lake ecosystems (Bradley 1999), and indirectly trigger oxygen changes in the water column and influence sedimentary processes.

Because of their peculiar chemistry, ferruginous lakes are used as analogues to Archean (4.0–2.5 Ga) and Paleoproterozoic (2.5–1.6 Ga) oceans, which are known for their anoxic and ferruginous conditions prior to major global oxygenation events (Lyons et al. 2014) leading to the deposition of iron formations worldwide. Understanding the redox picture of ancient iron formations and the biogeochemical cycles in modern analogues contribute to answering major questions regarding global oxygenation, the evolution of life and past climate changes (Asael et al. 2013; Swanner et al. 2020).

Given the high reactivity of iron to oxygen, iron minerals forming in the water column are potential indicators of basin-redox conditions. Therefore, many paleoclimate studies have widely used Fe speciation to reconstruct ancient and recent water-column dynamics (Lyons and Severmann 2006; Johnson et al. 2008; Reinhard et al. 2013). In particular, the isotope signature of Fe minerals (especially the ratio ^56^Fe/^54^Fe) has significantly assisted at constraining aquatic and subaquatic redox processes (Johnson et al. 2003; Lyons and Severmann 2006; Reinhard et al. 2013). These signatures are characterized by large Fe isotopic variability (*δ*^56^Fe of −3 to 1‰) in anoxic sediments and chemical precipitates, as opposed to near zero *δ*^56^Fe values in clastic sediments from oxic environments (Beard and Johnson 2004). Indeed, redox processes remobilizing iron, whether they are abiotic (Anbar et al. 2000; Bullen et al. 2001) or biologically mediated (Johnson et al. 2004), are usually characterized by reduced phases enriched in the light isotopes. Conversely, heavy Fe isotopes often accumulate in ferric minerals primarily attributed to kinetic isotope fractionation during the formation process (Johnson et al. 2004).

Redox-boundary processes have been identified and described in lacustrine and marine sediments using Fe isotope ratios (Teutsch et al. 2009; Busigny et al. 2014; Liu et al. 2015; Dauphas et al. 2017). In ferruginous stratified lakes with a seasonal or persistent chemocline, the partial oxidation of aqueous iron can lead to the formation of pelagic Fe oxyhydroxides (Song et al. 2011; Ellwood et al. 2019; Zheng et al. 2019). As a result, the aqueous phase is often enriched in the light isotope, with *δ*^56^Fe values down to − 2‰ (Malinovsky et al. 2005; Teutsch et al. 2009; Busigny et al. 2014). Preferential incorporation of the light isotope in the solid phase is well known in ferrous solid phases such as pyrite (Rolison et al. 2018). A few studies, however, showed a preferential incorporation of light Fe isotopes in ferric minerals: in the Baltic Sea, Fe oxyhydroxides with *δ*^56^Fe values of − 0.6‰ were interpreted as precipitating at a fast rate owed to increased reaction kinetics (Skulan et al. 2002). In Aha Lake (China), Fe oxyhydroxides with negative Fe isotopic composition (− 1.36 to − 0.10‰) resulted from persisting dissolution and precipitation processes during the setting of a seasonal chemocline (Song et al. 2011). In that study, suspended particulate matter (SPM) showed negative values principally due to allochthonous material with already low *δ*^56^Fe values (− 0.88 to + 0.07‰) carried into the lake from a highly weathered pyrite and coal-bearing catchment. In Hongfeng Lake (China), low *δ*^56^Fe values in SPM were explained by absorption of light iron by algae (Zheng et al. 2019). In summary, the Fe isotopic composition of authigenic ferric minerals depends considerably on the isotopic composition of the initial detrital material, basin dynamics (i.e., absorption, dissolution and precipitation processes, water–column mixing) and precipitation kinetics.

Even though processes occurring in the water column generate a wide range of isotopic variability, *δ*^56^Fe values of sediments might not reflect such processes. Early diagenesis can remobilize iron and form secondary minerals (Severmann et al. 2006). For instance, under anoxic conditions, ferric iron can be microbially reduced. The dissimilatory reduction of Fe^3+^ to Fe^2+^ (DIR) is known to fractionate iron in a range of up to 3‰ (Crosby et al. 2005, 2007; Teutsch et al. 2009; Liu et al. 2015). Reduced iron with negative *δ*^56^Fe values is carried back to solution (Fe^2+^_aq_) and can eventually form a variety of secondary Fe minerals (e.g. carbonates, sulfides, phosphates, divalent Fe oxides), depending on the redox conditions, pH and reduction rates (Zachara et al. 2002). In the context of quantitative Fe^3+^ reduction, as observed in the sediments from Lake Geneva (Switzerland) (Percak-Dennett et al. 2013), no detectable isotopic fractionation was found in their Fe isotope ratios. In such cases, the entire iron pool is transferred from one phase to another without significant fractionation. A similar scenario is seen in the siderite concretions of the Mazon Creek fossil site (309–307 Myr, Illinois) where near-zero δ^56^Fe values are reported (McCoy et al. 2017) with total consumption of microbially produced Fe^2+^ during the formation of solids. However, it is important to consider that isotopic fractionation in precipitated ferrous Fe would only occur if the whole ferric Fe pool was initially reductively mobilized on a quantitative scale. In the absence of quantitative reduction, the fractionation signal from microbially mediated DIR may still be apparent in the solid-phase ferrous Fe. Thus, diagenetic processes have the potential to imprint or even reset the isotopic range, which can influence interpretations related to past water–column-redox conditions and lake dynamics.

Despite some cases where there is nearly total Fe^3+^ reduction or Fe^2+^ precipitation, low isotope variability in basins generally supports an isotopic signature dominated by the Fe influx from the catchment (Staubwasser et al. 2013). Inversely, high variability characterizes intense reduction of Fe oxides and Fe (oxy) hydroxides, generating a Fe^2+^_aq_ pool with negative *δ*^56^Fe values. The dissolved Fe^2+^_aq_ pool eventually fuels the production of Fe^2+^ phases preserving negative *δ*^56^Fe signatures via partial precipitation (Song et al. 2011; Busigny et al. 2014). Therefore, low *δ*^56^Fe values can be expected at high concentrations of dissolved iron in basins where the Fe^3+^ substrate is abundant (Staubwasser et al. 2013).

Many iron remobilization processes may not be recorded after sediment deposition (Percak-Dennett et al. 2013; McCoy et al. 2017). Thus, understanding the cryptic cycle of iron is crucial for paleoclimate reconstructions. In this study, we focused on the redox front of Lago Fagnano (Argentina/Chile), a well-oxygenated lake with previous sedimentary and paleoclimatic studies (Moy et al. 2011; Waldmann et al. 2011, 2014; Vizcaino et al. 2023). For the first time we measured Fe-isotopes at 1 cm resolution in a 40 cm sediment profile of this lake. Our hypothesis is that the oxidation of Fe^2+^_aq_ below the sediment–water interface (SWI) into Fe (oxy)hydroxides, and subsequent dissimilatory iron reduction (DIR), create a dynamic cycle where light iron is constantly recycled (as described by Song et al. 2011). We argue that Fe isotope variability is recorded under specific circumstances, such as changes in sedimentation rates (Neugebauer et al. 2022) and turbiditic deposition (Waldmann et al. 2011). These interruptions in the Fe isotope recycling system promote the preservation of ferric-enriched mineral layers.

**Fig. 1:**
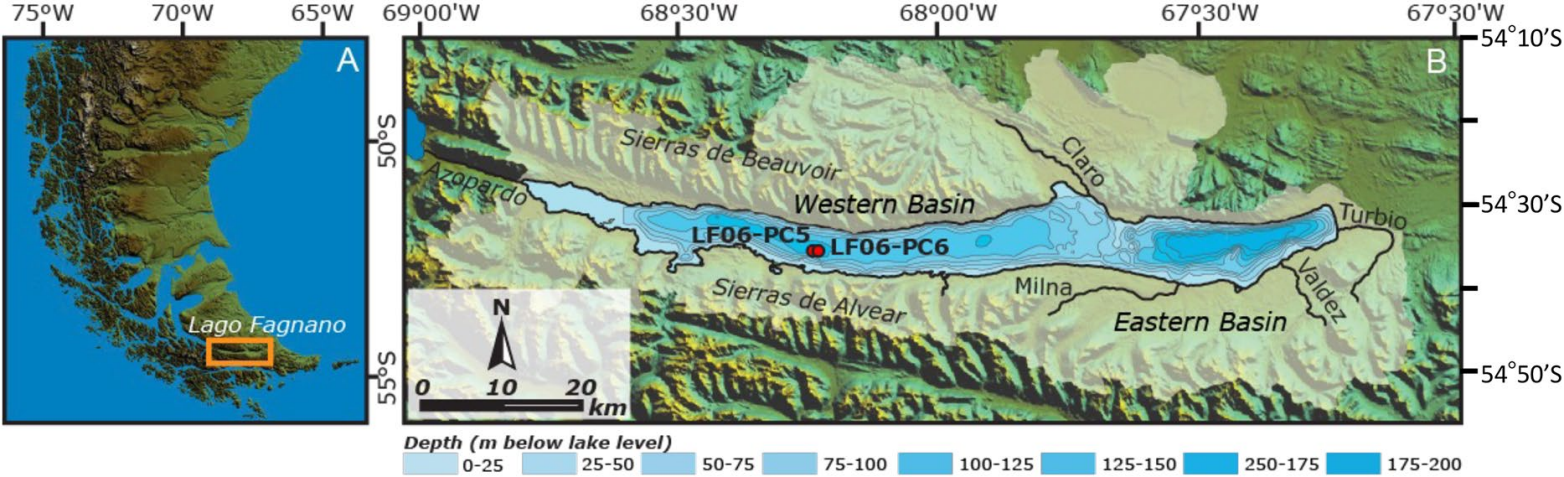
**A** Location of Lago Fagnano in Tierra del Fuego. **B** The Lago Fagnano watershed (white area) drawn on a SRTM map of the region. The two cores used for this study are marked: LF06-PC5 and LF06-PC6

Lago Fagnano (54° S, 67° W, 140 m a.s.l.) is a tectonic and glacial lake located in Tierra del Fuego, between Argentina and Chile (Fig. 1). It is the southernmost ice-free lake outside of Antarctica and, as such, a gateway to understand past and present relations between Antarctic, South Atlantic and South Pacific climate systems (Waldmann et al. 2014). The lake is divided into a smaller eastern sub-basin with a maximum water depth of 210 m and a larger and shallower western-central sub-basin of maximum 130 m water depth (Fig. 1). Water–column profiles from November 2006 in the eastern and western sub-basins indicate no apparent thermoclines and oxygen concentrations show little variations (from ca. 10 to 13 mg/L) indicating oxic conditions all through (Waldmann et al. 2014). The presence of broken littoral diatom frustules across some sediment cores (Waldmann et al. 2014) suggests a past persisting wind-driven wave activity and lake mixing with recycling of sediments from shallower parts that are transported into the deep basin (Neugebauer et al. 2022). Sediments exhibit a cyclic alternation of light gray and black to occasionally dark green laminae. These dark laminae were described through high resolution XRF scanning and appear to be enriched in Fe oxides and amorphous Fe monosulfides (Neugebauer et al. 2022).

Initially, the formation of laminations in Lago Fagnano was interpreted to be a result of changes in the water-column-redox conditions (Waldmann et al. 2014). However, recent research on the same cores suggests an alternative explanation for the lamination formation. It has been proposed that rapid increases in sedimentation rates lead to the burial of redox fronts, giving rise to the observed laminated sediments (Neugebauer et al. 2022). Under conditions of high sedimentation rates, there is a rapid influx of sedimentary material, which can overwhelm the rate of organic matter decomposition and the supply of oxygen from the water column. As a result, redox–sensitive metals and minerals, such as iron in the form of ferric (Fe^3+^) minerals, may become rapidly buried and preserved within the sediment layers. This burial effectively isolates the ferric minerals from further reactions with dissolved components in the water column, preserving their initial isotopic composition. Conversely, under low sedimentation rates, there is a slower accumulation of sedimentary material. This slower deposition allows for more interaction between the water column and the sediment, which may lead to the reworking and redistribution of redox–sensitive minerals. In this scenario, the isotopic signature of the ferric minerals may become less distinct as they undergo more dynamic exchanges with the surrounding environment. Therefore, the type of sediment deposited and preserved in Lago Fagnano appears to be closely linked to the sedimentation regime. High sedimentation rates favor the preservation of distinct laminated layers enriched in ferric minerals, while low sedimentation rates may result in a more homogenized distribution of isotopic signatures due to increased interaction and exchange between the water column and the sediment.

## Material and methods

Cores LF06-PC5 and LF06-PC6-4/4 (hereinafter referred to as PC5 and PC6), with lengths of 212 and 100.5 cm, respectively, were both taken in 2006 in the western basin of Lago Fagnano (Fig. 1) at a depth of 126 m. The cores were stored airtight at 4 °C in a cooling facility at the University of Geneva. Sediment features and mechanisms for the formation of laminae were previously described in core PC5 (Neugebauer et al. 2022). Sequential extractions of Fe phases and Fe isotope measurements in this study were performed in core PC6. Although the outer ca. 1 mm of the core was heavily oxidized (and avoided during sampling), the fine dark lamination characterizing these sediments was not oxidized and perfectly preserved. Despite that the sampling was not done under an oxygen–free environment, sediment digestion was made immediately in order to prevent both oxidation and isotope fractionation for the extraction products.

Following a sequential extraction procedure (Poulton and Canfield 2005), we aim at quantifying the most reactive Fe phases described in Lago Fagnano, i.e., Fe oxyhydroxides and Fe monosulfides (Neugebauer et al. 2022), and determine their Fe isotope signature. Indeed, while poorly reactive Fe phases are less sensitive to redox reactions and thus, to isotopic fractionation, Fe phases forming in the water column can potentially record lake dynamics and water-redox conditions at the time of mineral formation. The first leaching, using 1 M Na-acetate, is efficient at dissolving carbonates (Tessier et al. 1979; Poulton and Canfield 2005), acid–volatile sulfides (AVS; Cornwell and Morse 1987; Henkel et al. 2016) and surface-reduced Fe^2+^ (Crosby et al. 2005, 2007; Henkel et al. 2016).

Since carbonates are absent in Lago Fagnano, we expect the recovery of amorphous Fe monosulfides and, to a lesser extent, adsorbed Fe. For instance, Na-acetate gave complete recovery of AVS from a fine-grained coastal sediment from Young Sound, NE Greenland (Poulton and Canfield 2005). To confirm this, we tested Na-acetate for the dissolution of 15 mg of pure crystalline Fe^2+^ sulfide (Sigma Aldrich), with only 21% of mineral dissolution. However, Heron et al. (1994) recovered 28% of crystalline FeS by Na-acetate (and full recovery by 0.5 M HCl), stating that samples with naturally less stable FeS should yield higher recoveries.

Forty samples from the topmost 40 cm of the core were collected right after core opening in continuous 1 cm steps on the working half of the core, from the central part of the sedimentary record using cut syringes (*ø* = 1 cm) in order to avoid contamination from the sides. Samples were weighted in the wet-state (ca. 0.4 g) and directly inserted in Na-acetate (first extraction step) to avoid oxygen exposure and prevent potential oxidation of very redox–sensitive Fe fractions during generally used freeze-dry-grinding steps. Moreover, poorer extraction efficiencies have been reported after freeze-drying, which has been shown to alter the initial chemical speciation contribution of heavy metals, particularly in the carbonate bound and exchangeable fraction (FeS in our case) (Zhang et al. 2001). We calculated the water content by taking additional samples from same depths as for sequential extraction and drying them overnight. Water content fluctuated between 44 and 58%. Duplicates were taken every 10 cm with a third replica close to the liner to constrain the oxidation effect.

A volume of 20 mL of 1 M Na-acetate adjusted to pH 4.5 with acetic acid was used for an average 210 mg of equivalent dry sediment. Reaction was performed in 50 mL centrifuge polypropylene tubes and shaken for 24 h at room temperature. After extraction, samples were centrifuged at 1600 g for 6 min and the supernatant was filtered through a 0.20 µm nylon/polyethersulfone filter. The precipitate was rinsed with 25 mL milli-Q water that was discarded after a second centrifugation.

The second extraction uses 1 M of hydroxylamine-HCl in 25% v/v acetic acid (Chester and Hughes 1967). It is designed for the dissolution of ‘easily reducible’ iron such as amorphous Fe (oxy) hydroxides (i.e., ferrihydrite and lepidocrocite) (Poulton and Canfield 2005; Henkel et al. 2016). Samples were leached and shaken in 20 mL reagent for 48 h, at room temperature, and processed in the same way as the first step of the sequential extraction. Previous studies (Poulton and Canfield 2005; Henkel et al. 2016) demonstrated that Na-acetate (pH 4.5) dissolved less than 2% of the Fe oxyhydroxides in sediments.

Between 10 (Na-acetate) and 50-fold (hydroxylamine–HCl) dilutions with ultra–pure 1% HNO_3_ were prepared to measure major and trace elements in leachates using a quadrupole ICP-MS at the University of Geneva, Switzerland. Multi-element standard solutions at different concentrations (0, 0.02, 1, 5, 20, 100 and 200 ppm) were used for calibration. For accuracy, the international TMDA 51.4 standard was measured and triplicates yielded a standard deviation for iron concentration (122 ± 5 ppm, 2SD) below 5% and well within the error of the reference material (118 ± 15 ppm, 2SD).

Afterwards, an oxidation routine was applied prior the acquisition of Fe-isotope ratios (Henkel et al. 2016) to avoid any interaction with the reagents and any undesired matrix-induced bias. Indeed, the presence of acids and reagents other than HNO_3_ may interact with MS analyses. Briefly, the matrix of the different Fe pools was broken down by oxidation, in a mixture of aqua regia (concentrated HNO_3_ and HCl, 1:3) with H_2_O_2_ following the method developed by Henkel et al. (2016). The process was repeated twice, and the second oxidation included heating at 120 °C for 12 h. Once the matrix is destroyed, a solution of NH_4_OH 25% and H_2_O_2_ was used to recover Fe as hydroxides. The precipitates were rinsed, centrifuged and re-dissolved in 8 N distilled HCl prior the application of ion-exchange chromatography following the method described by Sheng-Ao et al. (2014). Moreover, ICP-MS analyses in the supernatant show an average iron recovery of 99.6% (Table S1, supplementary material).

For Fe isotope analyses, a Bio-Rad AG-MP-1 M strong anion exchange resin (100–200 mesh; chloride form) was used to separate Fe from the matrix elements. The resin was rinsed ten times with milli-Q water and adjusted to 2 mL in pre-cleaned Poly-Prep columns. The resin was further washed with 0.5 N HNO_3_ and 8 N HCl alternating with milli-Q water three times, then conditioned with 8 N distilled HCl following the procedure of Sheng-Ao et al. (2014). Prior loading, samples were diluted in 1 mL 8 N HCl to match a Fe concentration of around 3.5 ppm. This concentration was previously tuned to produce ideal signals of 35–45 V for the isotope ^56^Fe (Poitrasson and Freydier 2005) on the low mass peak shoulder. Matrix elements (﻿e.g. Na, Mg, Al, K, Ca, Ti, Cr, Ni and Mn) were eluted and discarded with the first 10 mL of 8 N distilled HCl, followed by Cu, with 24 mL of the same solution. Iron was recovered with 20 mL of 2 N distilled HCl. A preliminary test showed complete recovery of iron with already 18 mL of acid. After recovery, samples were evaporated and re-dissolved in 2% HNO_3_ for analysis.

Fe isotope analyses were performed on a Neptune Plus MC-ICP-MS at the University of Geneva, Switzerland. We followed a standard-sample bracketing calibration method using the IRMM-524a international reference material, which has nearly identical values (δ^56^Fe =  − 0.001 ± 0.013 ‰; Craddock and Dauphas 2011) to the widely used and discontinued IRMM-014. Fe isotope measurements were performed with standard Ni cones in a medium mass resolution mode (Poitrasson and Freydier 2005) on the low mass peak shoulder to avoid the mass interferences of isobaric polyatomic ions (Weyer and Schwieters 2003), using a standard glass spray chamber. Polyatomic interferences of ^40^Ar^14^N, ^40^Ar^16^O, ^40^Ar^16^O^1^H, ^40^Ar^18^O with the different Fe isotopes (^54^Fe, ^56^Fe, ^57^Fe, ^58^Fe, respectively) produced intensities below 1 mV, except for ^40^Ar^16^O which intensity was 2–3 mV. These intensities are < 0.01% than the sample signal corresponding. Potential interferences of ^54^Cr on ^54^Fe and ^58^Ni on ^58^Fe were monitored at masses ^52^Cr and ^60^Ni and corrections to Fe isotope ratios were made. Samples were analyzed between three to five times (with 40 bracketed measurements, or cycles, for each sample), in series of 40 samples in two different sessions. Four international rock standards (BHVO, AGV-2, JA-2, 08-BI-12; Czaja et al. 2013; He et al. 2015) were as well processed by anion-exchange chromatography. They were measured up to 11 times, discontinuously, in four different sessions and yielded excellent external reproducibility, well within the error (Table S1). Fe isotope results are reported relative to the IRMM-524a, using the standard delta notation (Equation 1):

1 $${\varvec{\delta}}^{56}\mathbf{F}\mathbf{e} \left[\mathrm{\permil }\right]=\left[(\phantom{l}^{56}{\varvec{F}}{\varvec{e}}/^{54}{\varvec{F}}{\varvec{e}} \, \mathbf{s}\mathbf{a}\mathbf{m}\mathbf{p}\mathbf{l}\mathbf{e}) / (^{56}{\varvec{F}}{\varvec{e}}/^{54}{\varvec{F}}{\varvec{e}} \, \mathbf{I}\mathbf{R}\mathbf{M}\mathbf{M}524\mathbf{a}) -1\right] \times 1000$$

## Results

### Fe and Mn concentrations and Fe isotopes

**Fig. 2:**
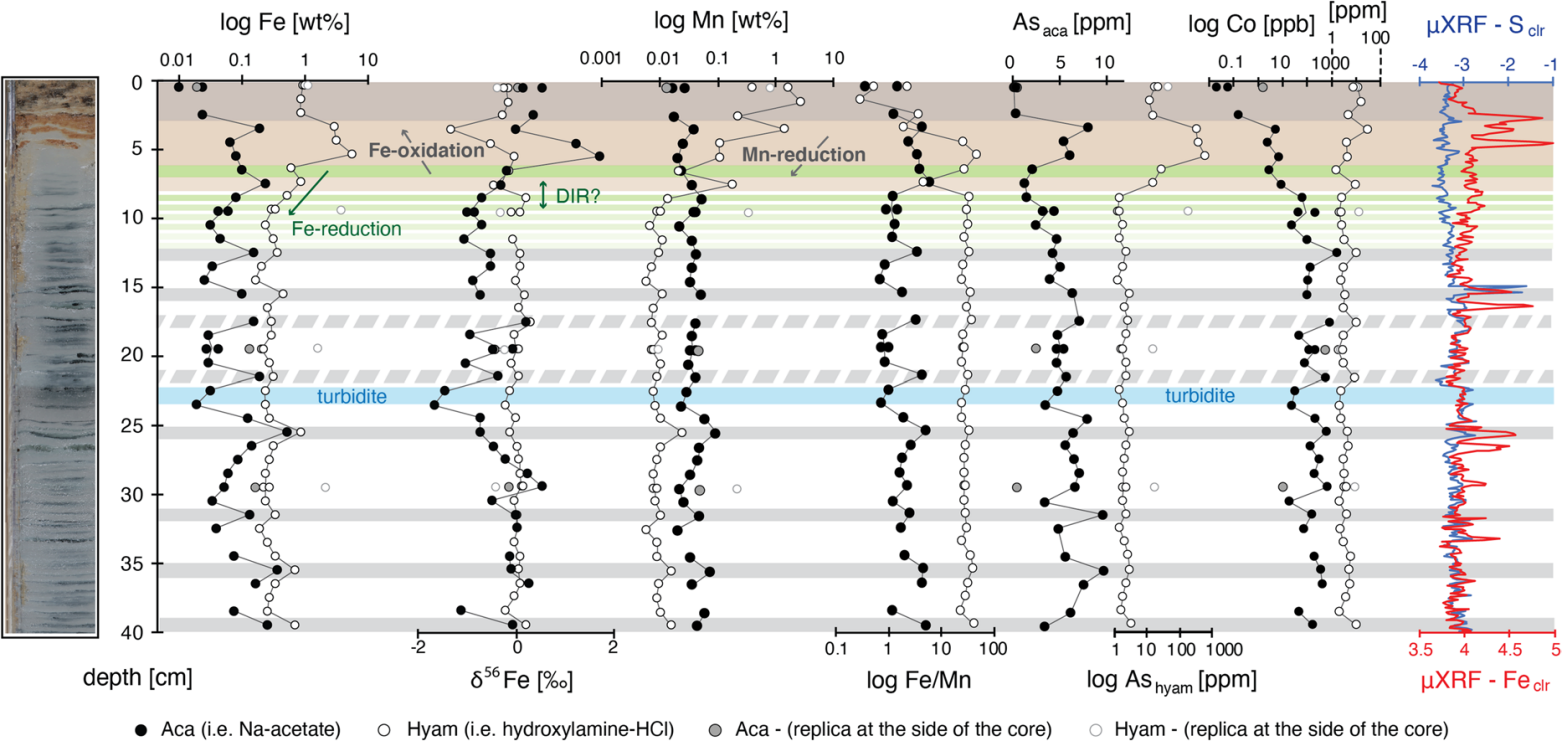
Chemical characterization of the uppermost 40 cm of core LF06-PC6. Major and trace elements obtained by ICP-MS analyses of leachates. Standard deviation (1SD) of Fe-isotope data is smaller than the symbols and thus not represented. μXRF profiles of bulk S and Fe given as centered log-ratios (clr) (Neugebauer et al. 2018). Solid gray bands likely represent paleoredox fronts and dashed gray bands show at least a redox transition, supported by Fe_hyam_ concentration peaks and high Fe/Mn ratios

The hydroxylamine-HCl reagent, targeting Fe oxyhydroxides, recovered variable Fe_hyam_ concentrations (averaging 0.63 wt%; Table S2). Maximal abundances are located between 3 and 6 cm depth (up to 5.5 wt%). Below 10 cm, Fe contents are nearly constant at ca. 0.30 wt% and disrupted by at least six peaks of up to 0.86 wt% of Fe_hyam_ (Fig. 2).

The Na-acetate solution, targeting reduced iron (Fe_aca_) phases, recovered an average of 0.10 wt% iron (Table S2). Eight high Fe concentrations peaks (up to 0.49 wt%) characterize the Fe_aca_ fraction, matching the same horizons of Fe_hyam_ (Fig. 2). The difference in concentration of both fractions is of up to two orders of magnitude at the top 6 cm of the profile and decreases with depth and at Fe-rich horizons.

**Fig. 3:**
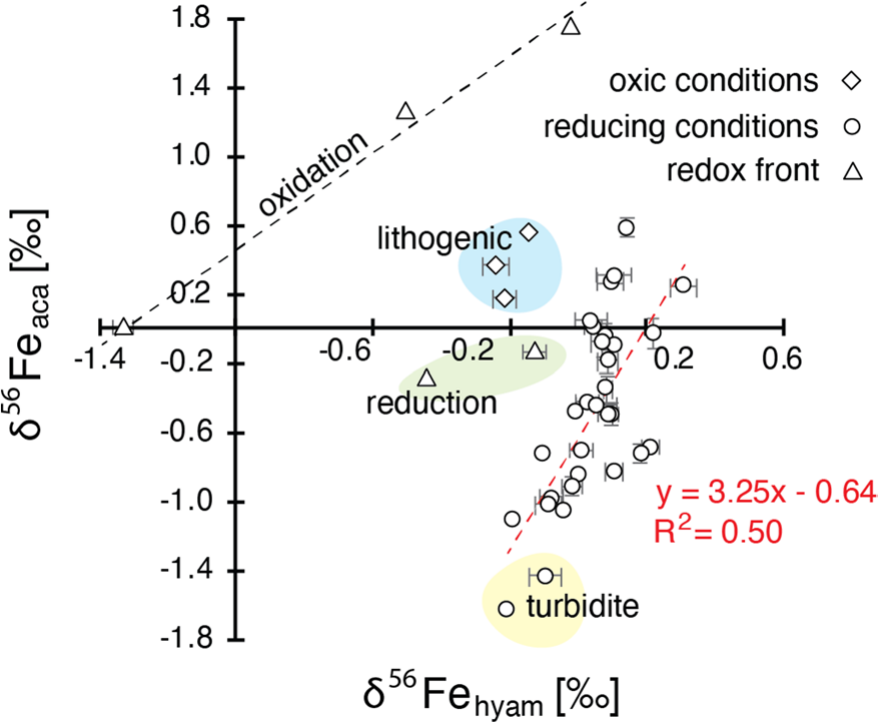
Fe isotopes measured above and below 8 cm depth (diamonds and circles, respectively) showing different correlation between the reduced and oxidized Fe phases (Fe_aca_ and Fe_hyam_, respectively). Bars represent 1SD. Between 4 and 6 cm (black diamonds), the positive relation between both Fe pools is linked to the consumption of dissolved Fe during Fe oxidation. Below 8 cm, δ^56^Fe_aca_ dispersion and low δ^56^Fe_hyam_ suggest two end-members. Negative δ^56^Fe_aca_ are typical of pore waters with a high amount of dissolved Fe (likely resulting from reducing conditions). Near zero values suggest low Fe reduction or near complete precipitation of dissolved Fe as reduced Fe phases

The *δ*^56^Fe_hyam_ range does not show high variability (− 0.03‰ ± 0.26‰), except between 3 and 5 cm depth, where *δ*^56^Fe_hyam_ reaches − 1.3 ‰ (Fig. 2). The *δ*^56^Fe_aca_ values are more variable along the profile and oscillate between − 1.6 and + 1.7‰. Below 8 cm depth, where values are more homogenous, the two fractions show a moderate positive correlation (*R*^*2*^ = 0.50; Fig. 3).

Even though the sequential extraction protocol is designed for Fe phases, Mn minerals are dissolved as well. In the upper 8 cm, Mn_hyam_ reaches much higher and variable contents (up to 2.9 wt%) than below (104 ± 36 ppm; Fig. 2). Conversely, Mn_aca_ contents are low and relatively constant along the profile (391 ± 163 ppm). The top 4 cm show low Fe_hyam_/Mn_hyam_ ratios (< 4, Fig. 2). Below, these ratios increase and reach an average value of ca. 29.5, except between 7 and 8 where the ratio drops to 4.5. Fe_aca_/Mn_aca_ values (averaging 2.3 ± 1.5) show a similar profile to Fe_hyam_/Mn_hyam_ (Fig. 2), although lower by one to two orders of magnitude. Generally, high Fe/Mn ratios are associated with Fe-rich peaks.

### Trace elements in the Na-acetate and hydroxylamine-HCl fractions

Many trace elements recovered with Na-acetate are relatively low in the topmost 8 cm. Elements such as As (averaging 4.9 ppm), Co (200 ppm), Sb (22 ppb) and Ni (26 ppb) increase with depth at different extents and positively relate with the reduced Fe_aca_ fraction (Fig. 2 and Fig. S1, supplementary material). However, some elements such as Mn (averaging 387 ppm; Fig. 2), Al (166 ppm), Cu (17 ppb) and Ba (1.5 ppm) do not show major variations throughout the core (Table S2).

The hydroxylamine-HCl leaching recovered abundant trace elements such as As_hyam_ (reaching 412 ppm, Table S2), V_hyam_ (6.7 ppm) and Ba_hyam_ (69.6 ppm), especially at the topmost 8 cm (Fig. 2). While As_hyam_ abruptly decreases below 8 cm, Sb and V concentrations slightly and progressively decrease with depth (Fig. S1). Among the measured trace elements, only Mg (averaging 260 ppm) and Zn (72 ppm) have lower values at the top of the profile, while Zn shows a peak (158 ppm) between 15 and 16 cm depth at which elevated S was detected by high–resolution XRF scanning (Fig. S1). Elements commonly associated with clastic fractions (Al and Ti) as well as Ni, Co, Cr, Cu and V do not show major stratigraphic variations in the hydroxylamine-HCl fraction (Table S2).

### Na-acetate and hydroxylamine-HCl leaching tests

Replicas close to the plastic core liner were retrieved every 10 cm to constrain post-coring oxidation effects in both fractions (Fig. 2). Any variation between replicas could not be identified at the top of the core (0–1 cm). At 10, 20 and 30 cm depth, replicas of the border, where a contact with oxygen is expected, generally have Fe_hyam_ contents ten times higher compared to those at the center (Table S2). Likewise, Mn_hyam_ contents at the border are multiplied by 3, indicating the potential of the hydroxylamine-HCl at further dissolving Mn^4+^ phases to form poorly ordered Mn oxides. Trace elements such as As_hyam_ or Sb_hyam_ are as well higher towards the liner. These observations support more oxidation at the sides but still reduced conditions in the center of the core.

Na-acetate leaching tests recovered more iron close to the liner suggesting a substantial contribution of Fe^3+^ phases (e.g. Fe^3+^ and/or mixed-valence (oxy) hydroxides) to the Fe_aca_ pool. Likewise, two fractions of As_aca_ with different oxidation states seem to be extracted with Na-acetate. The first, relatively abundant at the top of the core (3–6 cm), may represent As bounded to Fe^3+^ phases. The second fraction, likely diagenetic, increases downwards below 8 cm depth. If Na-acetate dissolves divalent Fe and As phases, it does not seem the case for Mn. Mn_aca_ recoveries (averaging 387 ppm) are generally similar, whether samples are at the center, sides or top of the core.

## Discussion

### Lago Fagnano sedimentary Fe-cycle

The limited geochemical information existing for the sedimentary profile of Lago Fagnano shows strong chemical differences. According to the trends defined by Fe_hyam_ and Fe/Mn ratios, it can be subdivided into two sections, with a clear transition at 8 cm depth (Fig. 2). This agrees with Neugebauer et al. (2022) who reported an active redox front a few cm below the SWI. The following 32 cm show a more homogenous chemical profile regularly disrupted by Fe and Fe/Mn peaks agreeing with Fe enrichment in both Fe pools.

In the topmost 3 cm, Fe_hyam_ (Fe^3+^) approaches 1 wt%. Right below, the Fe_hyam_ content reaches its maximum (5.5 wt%). Here, Fe enrichment can be explained by the formation of Fe-oxyhydroxides at a redox boundary. This is supported by Neugebauer et al. (2022) who showed (by *μ*XRF) higher Fe/Al ratios than elsewhere (Fig. S2). Below this redox boundary, where Fe reduction is expected, the Fe_hyam_ fraction decreases dramatically and stabilizes at ca. 0.3 wt% until the bottom of the core. Remnant Fe_hyam_ can be preserved if organic matter is a limiting factor for dissimilatory Fe reduction (Zhu et al. 2021).

The Fe_aca_ (averaging 965 ppm) fraction is low at the top of the core and higher below, where Fe^2+^ is more stable. This agrees with the presence of acid-volatile sulfides and surface-reduced Fe^2+^ in this fraction. However, dissolution tests at the border of the core, rich in iron oxides, indicate possible contribution of Fe^3+^ phases in the Fe^2+^ fraction. Despite this, the production of early diagenetic Fe^2+^ phases can be inferred from the top-down increase of trace elements such as As_aca_, Co_aca_, Ni_aca_ and Sb_aca_ (Fig. 2 and Fig. S1), commonly found in Fe (mono) sulfides (Huerta-Diaz and Morse 1992; Huerta-Diaz et al. 1998). Alternatively, a chemocline defining slightly sulfidic to euxinic conditions at the bottom of the water column would allow the production of pelagic Fe-(mono) sulfides (Busigny et al. 2014; Ellwood et al. 2019). Considering the lack of evidence of past water-column stratification (Waldmann et al. 2011), the formation of reduced Fe phases in the open water can be excluded.

Near zero isotope values of the Fe_hyam_ fraction above 3 cm and below 8 cm is as well consistent with the isotopic signature of allochthonous Fe phases (Beard and Johnson 2004). Within the redox front (3–8 cm) the low *δ*^56^Fe_hyam_ values might be linked to the formation of Fe-oxyhydroxides. Moreover, *δ*^56^Fe_hyam_ and *δ*^56^Fe_aca_ values correlate positively and suggest an isotopic relation between Fe oxyhydroxides and Fe^2+^ phases (Fig. 3) in which one phase is the byproduct of the other. *δ*^56^Fe_aca_ values are highly variable (Fig. 3) showing two main endmembers. Pore-water data are unfortunately not available for our site, but it has been shown that diagenetic phases with negative *δ*^56^Fe values can form in pore waters with a high amount of dissolved iron (i.e., under reducing conditions) that is probably the case in this lake. When close to zero these values suggest low Fe reduction or near complete precipitation of dissolved Fe as reduced Fe phases.

### δ^56^Fe values in the active redox front

**Fig. 4:**
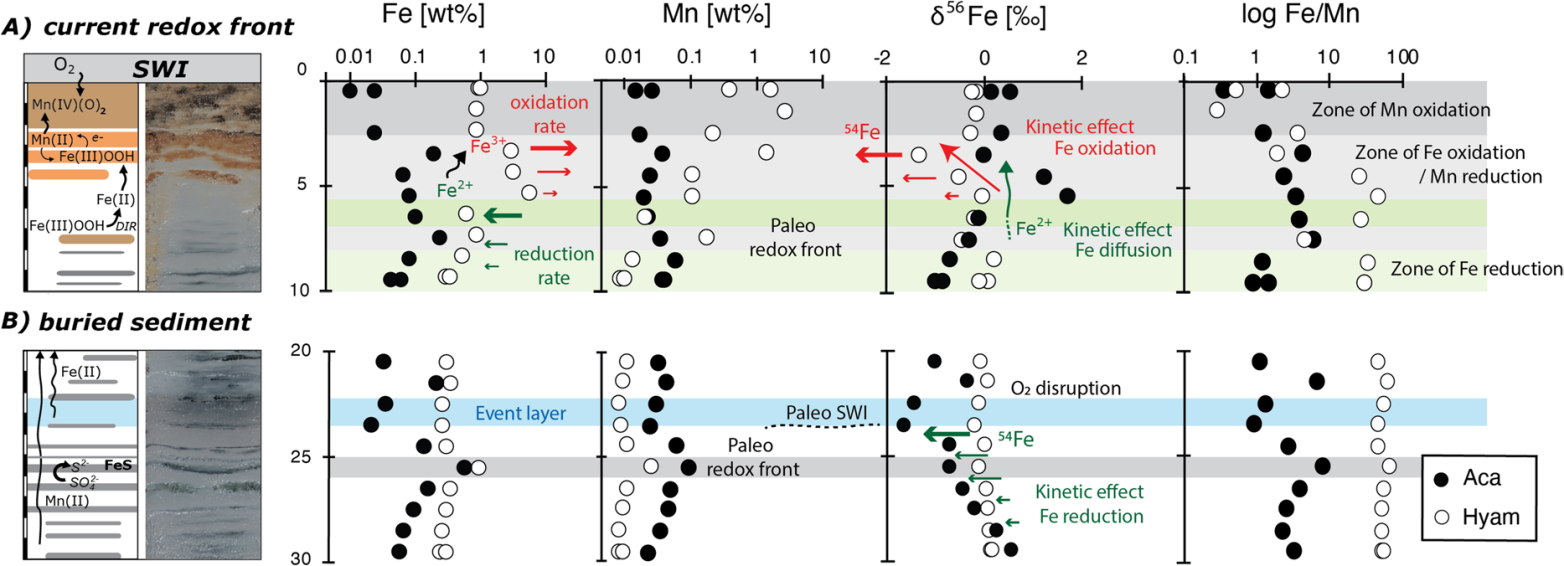
(A) Active redox front: From the sediment–water interface down to 3 cm depth, high contents of Mn_hyam_ indicate oxidation of Mn^2+^. Between 3 and 4 cm, Fe oxyhydroxides (Fe_hyam_) with very low δ^56^Fe values agree with kinetic fractionation likely as a kinetic effect. Between 5 and 6 cm, much higher amounts of Fe (oxy) hydroxides with near zero δ^56^Fe values possibly indicate near quantitative oxidation of aqueous Fe. The decrease in the Mn_hyam_ fraction suggests that Fe oxidation is coupled to Mn reduction. Most of Fe reduction occurs below 8 cm depth. (B) Buried redox front: High amounts of Fe (oxy) hydroxides below 25 cm covered by a turbiditic event suggests the preservation of a redox front. The partial reduction of ferric phases generates light Fe^2+^_aq_ which following O_2_ disruption relocates in the Fe_aca_ fraction (e.g., adsorbed Fe^2+^, amorphous Fe monosulfides and Fe oxides). Solid circles refer to the Na-acetate extraction (aca) whereas open circles to hydroxylamine-HCl. Standard deviation (1SD) of Fe-isotope data is smaller than the symbols and thus not represented

The main Fe oxidation zone (3–6 cm) is characterized by high Fe_hyam_ (3 to 5.5 wt%) and significant *δ*^56^Fe_hyam_ variability. The *δ*^56^Fe_hyam_ is maximal (0‰) at the base of the oxidation zone (5–6 cm) and minimal (− 1.3‰) at the top (3–4 cm) (Fig. 4A). The same feature appears in other lacustrine systems, such as at the redox boundary of Lake Kutsasjarvi (Sweden), where Fe oxidation occurs between 8 and 11 cm below the SWI (Malinovsky et al. 2005). In that study, Fe oxides at 11 cm depth constitute more than 69% of the bulk iron and have a *δ*^56^Fe of + 0.2‰, while at 9 cm Fe oxides drop to 32% and their *δ*^56^Fe values decrease to − 0.8‰.

High isotope values in Fe oxides and Fe oxyhydroxides have been widely studied and are the result of a two-step process. First, the fractionation linked to the oxidation of Fe^2+^_aq_ to Fe^3+^_aq_ under equilibrium conditions (Δ^56/54^Fe _Fe_^3+^_aq–Fe_^2+^_aq_) with values of up to + 3‰ in the ferric phase (Johnson et al. 2002; Crosby et al. 2005). Second, the formation of Fe oxyhydroxides, which follows an equilibrium or kinetic fractionation depending on the precipitation rates of Fe^3+^_aq_. Low rates result in Δ^56/54^Fe_hematite_–_Fe_^3+^_aq_ of 0.10‰ ± 0.20‰ whereas high rates reach a fractionation of − 1.32‰ ± 0.12‰ (Skulan et al. 2002). At the end, the entire process generally results in Fe^3+^ minerals with positive *δ*^56^Fe values, between 1 and 2‰ higher than Fe^2+^_aq_ as already confirmed by several studies in natural systems (Bullen et al. 2001; Severmann et al. 2006; Staubwasser et al. 2013). However, our results show that Fe^3+^ phases (Fe_hyam_) at the oxic-anoxic boundary have *δ*^56^Fe values 1 to 1.7‰ lower than the reduced fraction (Fe_aca_).

Lower isotopic values in the oxidized phase were already shown in other studies describing the formation of authigenic Fe oxyhydroxides with negative *δ*^56^Fe values as a consequence of a kinetic effect during mineral precipitation (Skulan et al. 2002; Staubwasser et al. 2013) or as resulting from persistent recycling of Fe following a chain of redox processes (Song et al. 2011). In our case, the isotopic balance of Fe_aca_ and Fe_hyam_ is close to zero everywhere along the core but the redox boundary. Since the Fe_aca_ fraction, isotopically heavier, is less abundant in this section, it cannot counterbalance alone the low isotopic values of the Fe_hyam_ fraction. Therefore, the range of *δ*^56^Fe values in this section must be controlled by kinetic effects triggered by the formation of Fe oxyhydroxides. This setting is consistent with an open system already enriched in the light isotope or a system in which a Fe-phase non-constrained in this study is enriched in the heavy isotope. This phase could be Fe_aq_. Although the isotope signature of the aqueous fraction was not constrained in this study, it can be deduced from the *δ*^56^Fe values of Fe_aca_, if an equilibrium hypothesis is made. That is, if the Fe_aca_ fraction is fueled by the leaching of authigenic phases such as FeS (e.g., mackinawite) and considering a Δ^56/54^Fe _FeS–Fe_^2+^_aq_ of + 0.3‰ (Wu et al. 2012), the theoretical isotopic signature of aqueous iron should be slightly lower below (− 0.3‰) than that of FeS phases.

The upward decrease in Fe_hyam_ and δ^56^Fe_hyam_ in the active redox front of Lago Fagnano (and Lake Kutsasjarvi; Malinovsky et al. 2005), is likely caused by the oxidation of Fe^2 + ^_aq_ transported from lower depths through pore-water diffusion (Davison 1993; Torres et al. 2014). Hence, the variation in the isotopic composition with depth can initially be attributed to a Rayleigh distillation effect occurring within the sediments. When the redox boundary is located in the chemocline, iron oxyhydroxides have the potential to form in the open water and are subsequently removed through a process known as decantation (Busigny et al. 2014). When the redox boundary is below the SWI, as it the case of Lago Fagnano, decantation cannot occur and pore-water diffusion plays a key role in the remobilization of iron. In ferruginous sediments charged in Fe^2+^_aq_, bottom-up diffused pore-water eventually meets the redox front and Fe oxyhydroxides can form (Bullen et al. 2001; Balci et al. 2006). Once oxidants are consumed, the Fe^2+^_aq_ in excess, enriched in the light isotope, continues the upwards diffusion and is oxidized with a lower isotopic signature. In addition, molecular diffusion can have a substantial kinetic effect in the fractionation of iron at this scale (LaBolle et al. 2008; Staubwasser et al. 2013; Druhan et al. 2019): a faster diffusion in the light Fe isotopes towards the top of the oxidation zone.

If the active redox front at the topmost 6 cm reveals Fe and Mn oxidation, redox processes such as Fe reduction are likely active at least down to 10 cm. Between 6 and 7 cm there is a sharp decrease of Fe_hyam_– and a slight decrease of *δ*^56^Fe_hyam_– (Fig. 4A), suggesting that most of the reduction occurs at this horizon. Normally, DIR promotes higher *δ*^56^Fe values in the solid phases and the production of Fe^2+^ with negative *δ*^56^Fe values (Crosby et al. 2005, 2007). At this stage, the formation of Fe^2+^ minerals and/or adsorption of Fe_aq_ could be expected. However, the scarcity of reduced Fe phases (i.e., Fe_aca_) with negative *δ*^56^Fe values are evidence for Fe^2+^_aq_ recycling towards the oxidation zone (Fig. 4A) as explained above. Alternatively, the production of sulfides via sulfate reduction could have enhanced the dissolution of ferric phases (Berner 1970). However, sulfate contents in Lago Fagnano are low and this process might be much more important below the active redox front, where partial pyritization during early diagenesis has been previously suggested (Neugebauer et al. 2022).

### Paleoredox fronts

It has been suggested that Fe-rich horizons are paleoredox fronts, preserved due to changes in sedimentation rates (Neugebauer et al. 2022). Under reducing conditions, Mn is more soluble than Fe (Burdige 1993; Davison 1993) and therefore, Mn and Fe/Mn ratios can be used to determine the onset of anaerobic sedimentary conditions as a result of oxygen depletion (Schaller et al. 1997). Here, Mn_hyam_ varies with ca. three orders of magnitude within the top 8 cm, with the highest values at the SWI (Fig. 4). Very low Fe/Mn occurs within the top 4 cm and overlies a nearly progressive increase until 8 cm. Both agree with the diagenetic reduction of substantial Mn^4+^ oxides coupled with Fe^2+^_aq_ oxidation (Dellwig et al. 2010; Song et al. 2011). This is particularly obvious between 6 and 8 cm, where low availability of Mn^4+^ phases (Mn_hyam_) can be tied to high contents of Fe oxyhydroxides (Fe_hyam_; Fig. 4A).

Redox boundaries are extremely dynamic and tend to migrate upwards as sedimentation takes progress. As a result, Fe-oxyhydroxides layers disappear with the reduction of iron. Occasionally, these redox fronts are preserved in the sediments under anoxic conditions. Dark green laminae in the Lago Fagnano core presented in this study are thin layers (1–2 mm) rich in Fe oxyhydroxides and have been suggested to represent paleoredox fronts (Neugebauer et al. 2022), comparable to those described in other similar settings (Granina et al. 2004; Och et al. 2012; Torres et al. 2014). A very likely mechanism responsible for the preservation of redox fronts is the increase of sedimentation rates (Granina et al. 2004), here suggested to be triggered by fast turbiditic deposition induced by earthquakes (Waldmann et al. 2011) and probably seasonal changes of increased run-off water flows (Neugebauer et al. 2022). In both scenarios, the water influx is altered promoting an acceleration in the sedimentation pattern, which consequently increases the supply of Fe.

A recent study carried out on another core retrieved from a sheltered area of the lake (Vizcaino et al. 2023) has also identified laminated sediments. These laminations were designated as hemipelagic sediments and considered to represent a fluctuating redox boundary in deep water conditions. Black laminae are preserved in an anoxic/dysoxic hypolimnion whereas light laminae are produced under increased oxic conditions. The authors came to the conclusion that, at least at this particular lake site known as Bahia Grande, water-column mixing is most likely driven by thermobaric instability during colder winters, and they linked ENSO—like conditions to the production of these laminations. Iron isotopes were not included in this investigation, though.

In the sedimentary cores that we studied, black laminae are thinner layers enriched in Fe sulfides and other trace metals due to reducing conditions and partial diagenetic pyritization. They occur below the redox front and at a higher frequency than green laminae. The sampling resolution in this study (1 cm) and the narrower thickness of black laminae (< ca. 1 cm) do not allow to distinguish geochemical variations between them. However, redox changes (i.e., high Fe_aca_/Mn_aca_) could be spotted at eight horizons while six of them present high contents of oxidized Fe phases (Fe_hyam_) and Mn oxides (Mn_hyam_) (Fig. 2), suggesting that at least six horizons could be considered as paleoredox fronts. Some of them are consistent with the high-resolution µXRF profile of bulk Fe (Fig. 2).

The Fe_hyam_ fraction decreases considerably below 10 cm and remains nearly constant below. Therefore, Fe reduction should not be a major diagenetic process in the anoxic sediments. Moreover, given the low *δ*^56^Fe variability in our dataset, DIR seems not to be the main process recorded in these sediments (Crosby et al. 2005). At the trace element scale, diagenetic remobilization of trace elements and the production of authigenic Fe phases can be deduced from relatively high Fe_aca_ values at Fe-rich horizons (Fig. 2). For instance, some trace elements like Sb and V decrease downwards in the hydroxylamine-HCl fraction while they increase (like As and Co) in the Na-acetate fraction and at Fe-rich horizons (Fig. S1). In addition, trace elements like As, Co and Ni support the production of diagenetic phases such as Fe monosulfides, as already reported in these (Neugebauer et al. 2022) and other sediments (Huerta-Diaz and Morse 1992; Huerta-Diaz et al. 1998). In particular, partial pyritization can be inferred from As enrichment in association with turbiditic events (Fig. 4B), which appear to still contain ferric phases or patches of Mn oxides scattered along the anoxic sediments (Neugebauer et al. 2022).

### Implications for paleoclimate studies

An increasing number of systematic Fe isotopic studies have been carried out recently in lacustrine sediments and authigenic minerals, which have been used for paleoclimatic reconstructions (Vuillemin et al. 2022). Considering the sampling resolution of our 40 cm core, ca. six paleoredox fronts could be identified thanks to their high content of bulk Fe and the Fe_aca_/Mn_aca_ relating Fe^2+^ and Mn^2+^. A horizon rich in iron and manganese is located at a depth of 25–26 cm beneath a turbidite layer that can be seen between 22 and 24 cm (Fig. 4B). Moreover, the isotopic trend in the reduced fraction (Fe_aca_) at the paleoredox front mirrors that of the oxidized fraction (Fe_hyam_) at the active redox front (i.e., a bottom-up enrichment in the light isotope). This suggests that in the studied cores the isotopically light Fe^2+^_aq_ resulting from DIR is preserved from degradation by oxidation following the turbiditic event that disrupts the oxygen flux and perpetuates anoxic conditions. As a consequence, Fe cycling is altered and reduced Fe phases with negative *δ*^56^Fe values can develop instead of Fe oxyhydroxides. This isotopic pattern is not visible in other paleoredox fronts suggesting that it is only after an abrupt change in the sedimentation rate that Fe isotope variations of redox boundaries can be preserved. This has important implications for paleoclimate studies and calls into question the isotopic record of redox processes in a long term, in situations in which sedimentation rates are constant. To elucidate this matter, additional research in these settings is required.

## Conclusions

Fe speciation and Fe isotopes measured in sediment cores from Lago Fagnano (southernmost South America) reveal the dynamics of the redox cascade and give insights on the active mechanisms associated with redox boundaries in a freshwater well–oxygenated lake. We were able to disentangle these processes at an active redox front and found a plausible basis for the nearly absent mid-term preservation of Fe redox signatures.

To summarize, the Fe cycling model proposed here comprises the following steps:

- In a stable ventilated Lago Fagnano, reduced light iron isotopes diffuse upward and oxidize at the SWI producing δ^56^Fe values in oxyhydroxides down to − 1.3‰, at the top of the redox front.
- Newly formed Fe oxyhydroxides are further reduced as sedimentation carries on at constant rates, resolubilizing light iron isotopes.
- If the sedimentation rate is constant, the redox front continuously migrates upwards. However, a considerable rapid increase of the sedimentation rate (e.g., turbiditic event) allows the preservation of a redox front and a light δ^56^Fe signature, in reduced Fe phases.

This model improves the understanding on how Fe signatures might be preserved in the paleorecord showing a typical signature of a redox boundary below a SWI. We further suggest that the proposed mechanism can be applied for understanding the geochemistry of earlier oceans.

## Supplementary Information

Below is the link to the electronic supplementary material.

Supplementary file1 (PDF 876 KB)

Supplementary file2 (XLSX 16 KB)

Supplementary file3 (XLSX 59 KB)
