# Supplementary figures and images for: Sediment-redox dynamics in an oligotrophic deep-water lake in Tierra del Fuego: insights from Fe isotopes
Source: J Paleolimnol. 2024 Apr 29;72(2):129–43. doi: 10.1007/s10933-024-00316-0 (PMC11576826; doi:10.1007/s10933-024-00316-0)

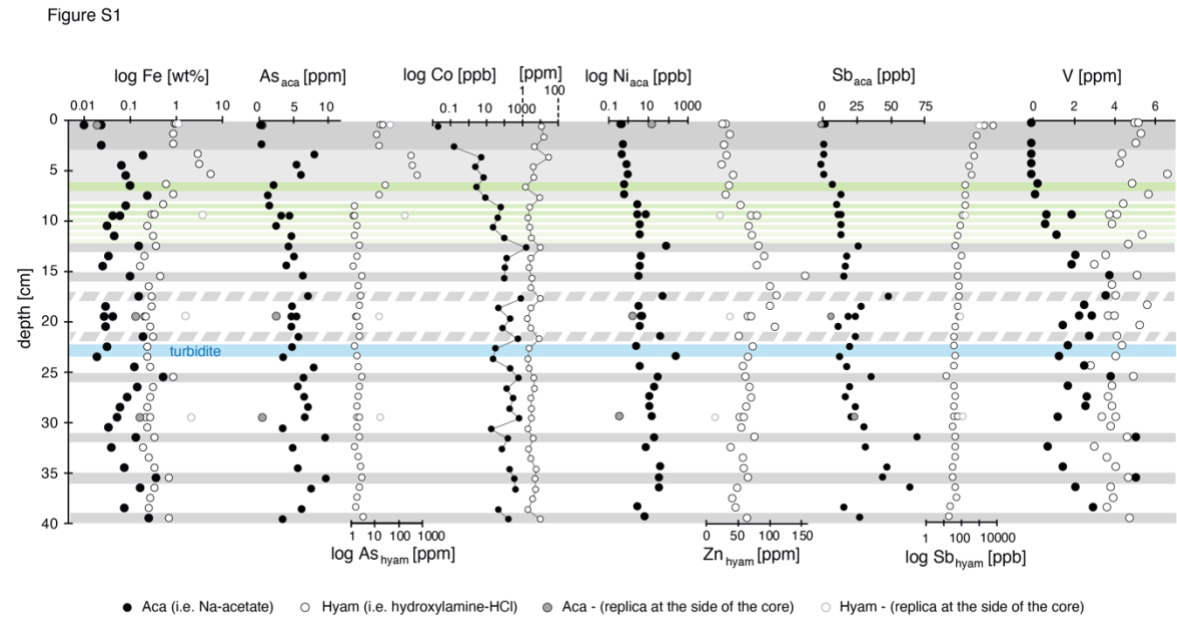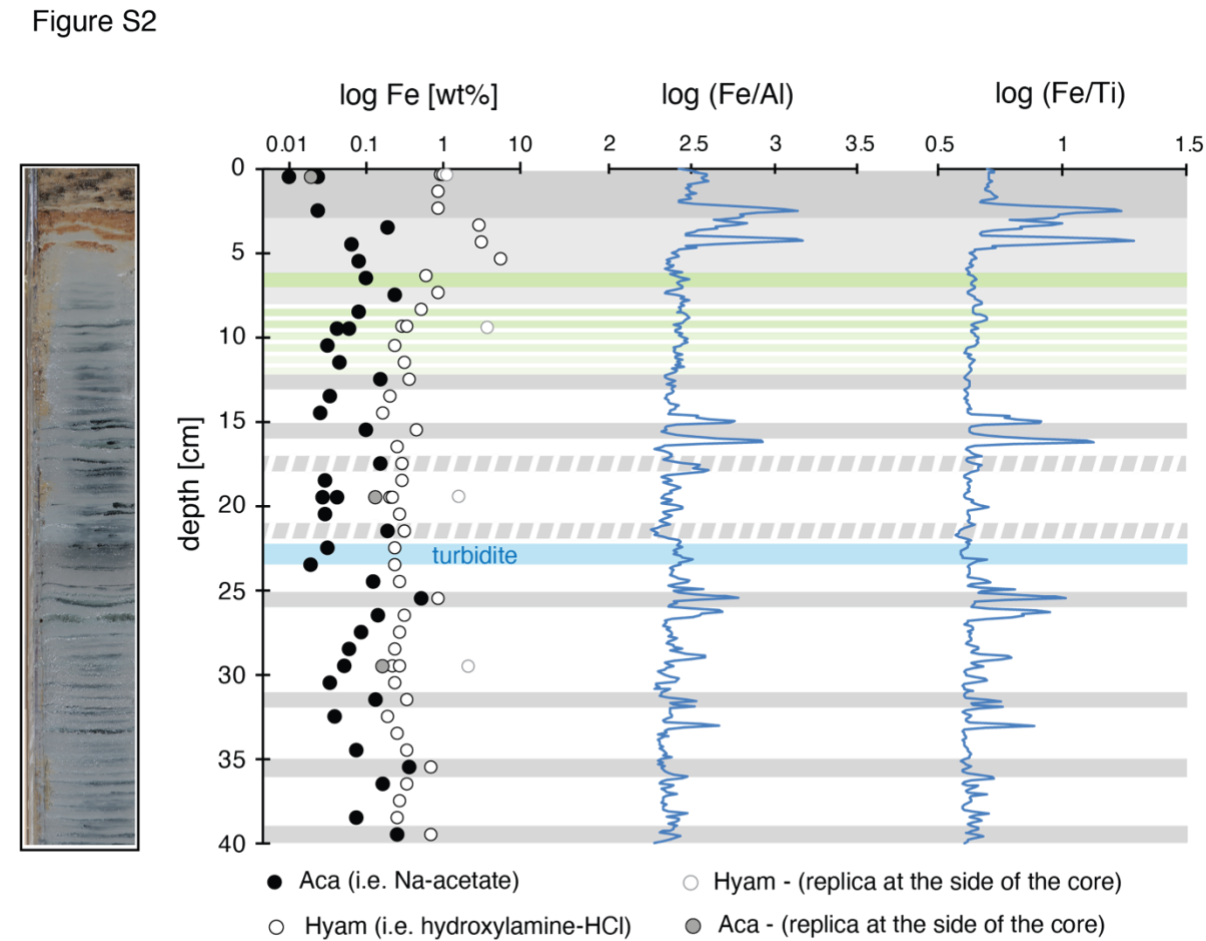

Supplement: Supplementary file 1 — Supplementary file1 (PDF 876 KB) [file 10933_2024_316_MOESM1_ESM.pdf]
